# Persistent but atypical germinal center reaction among 3^rd^ SARS-CoV-2 vaccination after rituximab exposure

**DOI:** 10.3389/fimmu.2022.943476

**Published:** 2022-08-10

**Authors:** Ana-Luisa Stefanski, Hector Rincon-Arevalo, Eva Schrezenmeier, Kirsten Karberg, Franziska Szelinski, Jacob Ritter, Yidan Chen, Christian Meisel, Bernd Jahrsdörfer, Carolin Ludwig, Hubert Schrezenmeier, Andreia C. Lino, Thomas Dörner

**Affiliations:** ^1^ Department of Rheumatology and Clinical Immunology, Charité Universitätsmedizin Berlin, Berlin, Germany; ^2^ Deutsches Rheumaforschungszentrum (DRFZ), Berlin, Germany; ^3^ Department of Nephrology and Medical Intensive Care, Charité Universitätsmedizin Berlin, Berlin, Germany; ^4^ Grupo de Inmunología Celular e Inmunogenética, Facultad de Medicina, Instituto de Investigaciones Médicas, Universidad de Antioquia (UdeA), Medellín, Colombia; ^5^ Berlin Institute of Health Charité Universitätsmedizin Berlin, Berlin Institute of Health (BIH) Academy, Berlin, Germany; ^6^ Rheumatology Outpatient Office RheumaPraxis Steglitz, Berlin, Germany; ^7^ Department of Medical Immunology, Charité University Medicine and Labor Berlin-Charité Vivantes, Berlin, Germany; ^8^ Institute of Transfusion Medicine, Ulm University, Ulm, Germany; ^9^ Institute for Clinical Transfusion Medicine and Immunogenetics, German Red Cross Blood Transfusion Service Baden-Württemberg – Hessen and University Hospital Ulm, Ulm, Germany

**Keywords:** rituximab (RTX), SARS-CoV-2, vaccination, memory B cell (MBC), germinal center (GC)

## Abstract

**Background:**

Durable vaccine-mediated immunity relies on the generation of long-lived plasma cells and memory B cells (MBCs), differentiating upon germinal center (GC) reactions. SARS-CoV-2 mRNA vaccination induces a strong GC response in healthy volunteers (HC), but limited data is available about response longevity upon rituximab treatment.

**Methods:**

We evaluated humoral and cellular responses upon 3rd vaccination in seven patients with rheumatoid arthritis (RA) who initially mounted anti-spike SARS-CoV-2 IgG antibodies after primary 2x vaccination and got re-exposed to rituximab (RTX) 1-2 months after the second vaccination. Ten patients with RA on other therapies and ten HC represented the control groups. As control for known long-lived induced immunity, we analyzed humoral and cellular tetanus toxoid (TT) immune responses in steady-state.

**Results:**

After 3^rd^ vaccination, 5/7 seroconverted RTX patients revealed lower anti-SARS-CoV-2 IgG levels but similar neutralizing capacity compared with HC. Antibody levels after 3rd vaccination correlated with values after 2nd vaccination. Despite significant reduction of circulating total and antigen-specific B cells in RTX re-exposed patients, we observed the induction of IgG+ MBCs upon 3^rd^ vaccination. Notably, only RTX treated patients revealed a high amount of IgA+ MBCs before and IgA+ plasmablasts after 3^rd^ vaccination. IgA+ B cells were not part of the steady state TT+ B cell pool. TNF-secretion and generation of effector memory CD4 spike-specific T cells were significantly boosted upon 3^rd^ vaccination.

**Summary:**

On the basis of pre-existing affinity matured MBCs within primary immunisation, RTX re-exposed patients revealed a persistent but atypical GC immune response accompanied by boosted spike-specific memory CD4 T cells upon SARS-CoV-2 recall vaccination.

## Introduction

Durable humoral immune responses to vaccination require generation of long-lived memory B cells (MBCs) and bone marrow plasma cells (BMPCs), commonly differentiating upon germinal center (GC) reactions (1). If circulating antibodies fail to confer protection to exposure, MBCs drive the recall response by forming new antibody secreting plasma cells or reentering germinal centers for additional rounds of somatic hypermutation (SHM) (2). SARS-CoV-2 mRNA 2x vaccination induces a strong GC response in healthy volunteers (3), but circulating antibodies are waning over time (4), emphasizing the role of persisting long-lived MBCs in combating breakthrough infections and preventing severe courses of the disease (5).

Rheumatologists are faced with several questions regarding the effectiveness and durability of SARS-CoV-2 vaccination responses particularly in patients receiving anti-CD20 therapy with rituximab (RTX). These patients are at higher risk for poor COVID-19 associated outcomes (6, 7) as well as substantially diminished SARS-CoV-2 vaccination responses (8–10) and a higher incidence of severe breakthrough infections after vaccination (11, 12). It is known that waning immunogenicity upon primary vaccination can be successfully boosted by a 3^rd^ homologous or heterologous mRNA vaccination associated with better COVID-19 outcomes in patients with autoimmune diseases (13, 14). However, longevity of immune responses in the context of RTX re-exposure still need to be delineated.

In this study we addressed the question about durability of vaccination-induced SARS-CoV-2 immune responses and induction of a secondary immune response in the context of restricted B cell availability upon rituximab re-exposure. Therefore, we assessed humoral as well as B and T cellular vaccination responses upon 3rd vaccination in RTX treated patients who initially mounted anti-spike SARS-CoV-2 IgG antibodies upon primary 2x vaccination (15) and got re-exposed to RTX 1-2 months after the second vaccination. As control for known long-lived induced immunity, we analyzed steady-state humoral and cellular tetanus toxoid (TT) immune responses.

## Materials and methods

### Study participants

RTX treated outpatients, who participated at our initial vaccination study (15) were screened for RTX treatment after the second vaccination. We identified 7 patients with rheumatoid arthritis [RA, according 2010 ACR Rheumatoid Arthritis Classification Criteria (16)], who received another course of RTX (1-2mg) treatment 1-2 months after the second SARS-CoV-2 vaccination. The patients were scheduled for a 3^rd^ vaccination with BNT162b2, 6 months after 2^nd^ vaccination according to federal state recommendations. We collected peripheral blood samples (EDTA anti-coagulated or serum-tubes, BD Vacutainersystem, BD Diagnostics, Franklin Lakes, NJ, USA) at 6 months (before 3^rd^ vaccination) and at d21 boost (3-4 weeks after the 3^rd^ vaccination). Ten RA patients receiving other therapies (RA group) and ten healthy controls (HC group) served as controls. TT antibody titers and antigen-specific B and T cells served as steady state control (last TT vaccination occurred 2-10 years before blood drawing). All participants gave written informed consent according to the approval of the ethics committee at the Charité University Hospital Berlin (EA2/010/21, EA4/188/20). Humoral vaccine response at d28 and d42 (**Figures 2A, C**), cellular data at d0 and d28 for total B cells, (**Figure 3A**), antigen specific (RBD+) B cells (**Figures 3D, E**) and at d28 for antigen specific T cells **Figures 5A, C, D**) have been partially previously published (15, 17).

### Enzyme-linked immunosorbent assay for SARS-CoV-2 and TT as well as surrogate SARS-CoV-2 neutralization test (GenScript)

The Euroimmun anti-SARS-CoV-2 assay is a classical enzyme-linked immunosorbent assay (ELISA) for the detection of IgG to the S1 domain of the SARS-CoV-2 spike (S) protein and IgG to the SARS-CoV-2 nucleocapsid (NCP) protein. The assay was performed according to the manufacturer´s instructions, as described (15). Briefly, serum samples were diluted at 1:100 in sample buffer and pipetted onto single wells of a 96-well microtiter plate, precoated with recombinant SARS-CoV-2 spike or nucleocapsid proteins. A calibrator, a positive control and a negative control were carried out on each plate. After incubation for 60 minutes at 37°C, wells were washed 3 times and the peroxidase-labelled anti-IgG antibody solution was added, followed by a second incubation step of 30 min. After three additional washing steps, substrate solution was added and the samples were incubated for 15 - 30 minutes in the dark. After adding the stop solution, optical density (OD) values were measured on a POLARstar Omega plate reader (BMG Labtech, Ortenberg, Germany) at 450 nm and at 620 nm. Finally, OD ratios were calculated based on the sample and calibrator OD values. An OD-ratio of ≥ 1.1 was considered to be positive for all analytes. IgG OD-ratio of ≥ 1.1 defines humoral seroconversion. Dilutions of 1:10 were prepared when values were close to the saturation point of the respective ELISA.

The Immunoassay for determination of TT (VaccZyme Tetanus-Toxoid IgG kit; The Binding Site) vaccine titer in serum was performed according to the manufacturers’ instructions. A level > 0.1 IU/ml is considered protective.

The blocking ELISA GenScript qualitatively detects anti-SARS-CoV-2 antibodies suppressing the interaction between the receptor binding domain (RBD) of the viral spike glycoprotein (S) and the angiotensin-converting enzyme 2 (ACE2) protein on the surface of cells, as described (17). Scores < 30% were considered negative, scores ≥ 30% were considered positive (linear quantitative range).

### Isolation of peripheral blood mononuclear cells and staining

PBMCs were prepared by density gradient centrifugation using Ficoll-Paque PLUS (GE Healthcare Bio-Sciences, Chicago, IL, USA). PBMCs before and after 3^rd^ vaccination were cryopreserved at -80°. Cells were thawed, washed twice in pre-warmed RPMI1640 medium [containing 0.3 mg/ml glutamine, 100 U/ml penicillin, 0.1 mg/ml streptomycin, 10% FCS and 25 U/ml DNase I (Roche International)] and stained as described (15). To identify RBD-specific and TT-specific B cells, respectively, recombinant purified RBD (DAGC149, Creative Diagnostics, New York, USA) and TT (peptides & elephants GmbH, Hennigsdorf, Germany) were labeled with either AF647 or AF488. Double positive cells were considered antigen-specific as reported. A blocking experiment using unlabeled RBD or TT respectively in 100-fold concentration was used to ensure specificity of detection as reported (15, 18). For intracellular staining after T cell stimulation cells were first stained for 30 min with 1:1000 BUV395 Life/Dead (Invitrogen) in PBS, followed by 5 min 2.5 µl Fc Block (Milteny Biotech) in 50 µl resuspended cells. Cells were fixed in LyseFix (Becton Dickinson), permeabilized with FACS Perm II Solution (Becton Dickinson) and intracellularly stained.

### Peptide stimulation for antigen specific T cells

2x10^6^ frozen PMBC from 8 HC, 8 RA control and 7 RTX were used per stimulation condition. Cells were thawed, washed twice in pre-warmed RPMI1640 medium (containing 0.3 mg/ml glutamine, 100 U/ml penicillin, 0.1 mg/ml streptomycin, 10% FCS and 25 U/ml DNase I (Roche International), rested for 1 h in culture medium (RPMI1640 with glutamine, antibiotics and 10% FCS) and stimulated with SARS-CoV2 spike (“PepMix” SARS-CoV-2 (S B.1.1.7), JPT, Berlin, Germany) or with TT peptide pool (peptides&elephants, Henningsdorf, Germany) or 50ng/ml PMA (SigmaAldrich) with 1μg/ml Ionomycin (SigmaAldrich) for 16 h. Brefeldin A (10 μg/ml, SigmaAldrich) was added after 2 h. Due to cell number limitations, T cell PMA/Ionomycin stimulation was not carried out for all participants. As previously shown, CD4 T cells co-expressing CD154 and CD137 were considered antigen-specific (15, 19). Spike-specific CD8 T cells were identified based on activation-dependent co-expression of CD137 and IFNγ. We defined responders as those with at least a twofold increase in frequency after stimulation compared with unstimulated controls.

### Flow cytometry analysis

All flow cytometric analyses were performed using a BD FACS Fortessa (BD Biosciences, Franklin Lakes, NJ, USA). To ensure comparable mean fluorescence intensities (MFIs) over time of the analyses, Cytometer Setup and Tracking beads (CST beads, BD Biosciences, Franklin Lakes, NJ, USA) and Rainbow Calibration Particles (BD Biosciences, Franklin Lakes, NJ, USA) were used. For flow cytometric analysis, the following fluorochrome-labeled antibodies were used: BUV737 anti-CD11c (BD, clone B-ly6), BUV395 anti-CD14 (BD, clone M5E2), BUV395 anti-CD3 (BD, clone UCHT1), BV786 anti-CD27 (BD, clone L128), BV711 anti-CD19 (BD, clone SJ25C1), BV605 anti-CD24 (BD, clone ML5), BV510 anti-CD10 (BD, clone HI10A), BV421 anti-CXCR5 (BD, clone RF8B2), PE-CF594 anti-IgD (Biolegend, San Diego, CA, USA, clone IA6-2), APC-Cy7 anti-CD38 (Biolegend, clone HIT2), PE-Cy7 anti-IgG (BD, clone G18-145), anti-IgA-Biotin (BD, clone G20-359), BV650 anti-IgM (BD, clone MHM-88), FITC anti-TNFα (Biolegend, clone Mab11), BV650 anti-IFNγ (BD, clone 4S.B3), BV786 anti-CD40L (Biolegend, clone 24-31), PE-CF594 anti-CD137 (Biolegend, clone 4B4-1). Numbers of absolute B and T cells were measured with Trucount (BD) and samples were processed according to the manufacturer’s instruction.

### Data analysis

All samples included in the final analyses had at least 1 × 10^6^ events with a minimum threshold for CD19+ cells of 5,000 events apart from RTX patients (minimal recorded CD19+ events in the RTX group were 24 and 30 events respectively, out of > 1 Mio total recorded events. Flow cytometric data were analyzed by FlowJo software 10.7.1 (TreeStar, Ashland, OR, USA). For UMAP analysis of antigen-specific CD19+ B cells flow cytometry data of all study participants was pre-gated on RBD+ and TT+ CD19+ B cells respectively, concatenated and clustered by CD27, IgD, CD38, CD24, IgM, IgG, IgA. As settings we selected the Euclidean distance function, nearest neighbor value of 15 and a minimum distance of 0.5.

### Statistics

GraphPad Prism Version 5 (GraphPad software, San Diego, CA, USA) was used for statistical analysis. Statistical analysis was conducted as indicated in respective figure legends. For longitudinal analysis Friedman test with Dunn´s post-test was applied. For group comparison Kruskal-Wallis with Dunn´s post-test was used. Wilcoxon matched-pairs signed rank test was applied for paired analysis. Correlation was evaluated using the Spearman test. P-values < 0.05 were considered significant.

## Results

### Cohorts and patient characteristics

**Figure 1:**
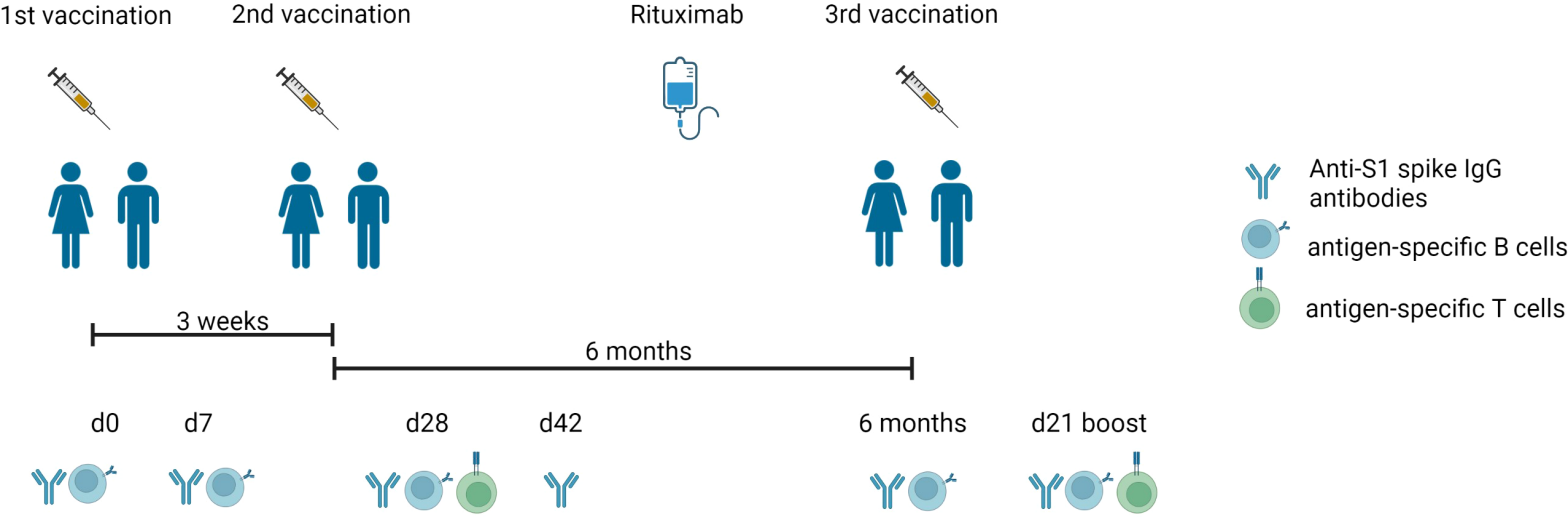
Experimental design of the vaccination study. Timeline describing the three-dose vaccination schedule of BNT162b2 mRNA vaccine, blood drawing (d0, d7, d28, d42, 6 months, d21 boost), and RTX treatment between 2^nd^ and 3^rd^ vaccination. Anti-S1 spike IgG antibodies, antigen-specific T and B cells were measured as mentioned. Created with BioRender.com.

We included 7 RTX treated RA patients, who received rituximab between 2^nd^ and 3^rd^ vaccination (RTX group), 10 RA patients on other therapies (RA group) and 10 healthy controls (HC group) in this study. In addition to the collected data before 3^rd^ vaccination (6 months) and 3 weeks after 3^rd^ vaccination (d21 boost), we also show data of a prior study (15) including d0 (before 1^st^ vaccination), d7 (7 days after 1^st^ vaccination), d28 (7 days after 2^nd^ vaccination) and d42 (3 weeks after 2^nd^ vaccination). The complete study design is summarized in **Figure 1**. The majority of study participants (22/27, 81,5%) were 3x-vaccinated with the mRNA vaccine BNT162b2. HC were younger (median 55 years old) than the patient groups (RA median 75 years old, RTX median 67 years old) and the majority of patients were female (HC 50%, RA 70% and RTX 100%, respectively). At the time of 3^rd^ vaccination, RTX patients had received B cell depleting therapy on average for 6.5 years and median time since the last RTX treatment was 5 months. Notably, 5/7 RTX treated patients did not have any treatment combination with disease-modifying antirheumatic drugs (DMARDs). Demographics and co-medication of all study participants are summarized in **Table 1** (detailed in **Supplementary Table S1**). To identify previously SARS-CoV-2 infected individuals or post-vaccination breakthrough infections, we measured antibodies against the nucleocapsid protein (NCP). Here 2 RTX patients were identified with positive anti-NCP IgG before first vaccination (**Supplementary Figure S1A** and as indicated in red in the manuscript figures), No breakthrough infections were recorded throughout the study in all groups (**Supplementary Figure S1A**).

**Table 1:** Patient characteristics.

	HC n = 10	RA n = 10	RTX n = 7
**Age**			
Median [IQR]	55 [41.25 – 61.5]	75 [65 – 79.5]	67 [63 – 73.5]
Under 40	3	1	0
Between 40-69	6	3	4
> 70	1	6	3
**Gender**			
Female	5	7	7
Male	5	3	0
**Vaccines (n)**			
3x BNT162b2	8	9	5
2x mRNA-1273, 1x BNT162b2	0	0	1
2x ChAdOx1, 1x BNT162b2	0	1	1
1x ChAdOx1, 2x BNT162b2	2	0	0
**Immunosuppression (n)**			
MTX		7	1
Leflunomid		1	0
Sulfasalazin		0	1
JAKI		2	0
TNFI		1	0
Abatacept		1	0
Prednisolone		1 (4mg/d)	1 (7.5mg/d)
**Months since last RTX**			
Median [IQR]			5 [5 – 6]
**Years on RTX**			
Median [IQR]			6.5 [2.75 – 6.5]

### Antibody levels after 2^nd^ vaccination predict humoral response upon 3^rd^ vaccination

**Figure 2:**
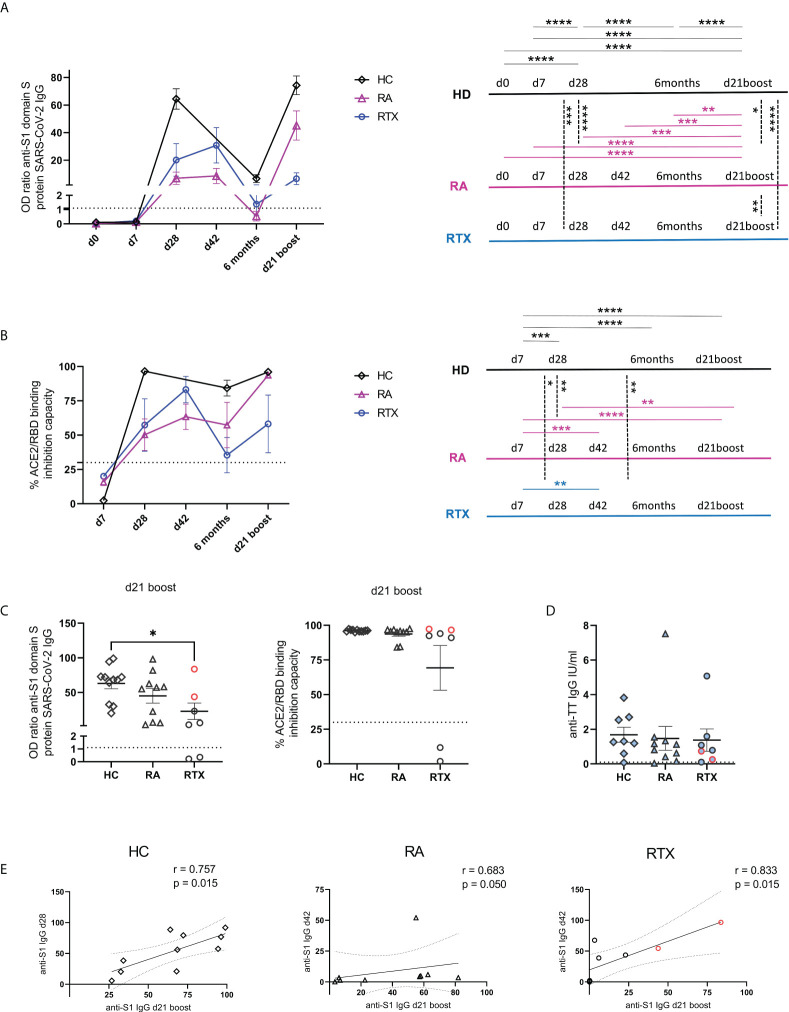
Trajectory of humoral response upon anti-SARS-CoV-2 vaccination. Humoral immune response against SARS-CoV-2 for spike protein S1 IgG **(A)** and surrogate neutralization **(B)** over time in HC (n=10), RA group (n=10) and RTX treated patients (n=5, the two pre-infected patients are excluded). Threshold for positive test is indicated by dotted lines. The results of the statistical tests are depicted on the right. Humoral immune response against SARS-CoV-2 for spike protein S1 IgG, surrogate neutralization **(C)** and anti-TT IgG titers **(D)** at d21 boost in HC (n=10), RA (n=10) and RTX (n=7). Correlation between anti-spike S1 IgG at d28 (HD)/d42 (RA and RTX) with anti-S1 levels at d21 boost **(E)**. Mean with SEM **(A-D)**. Two way ANOVA with Šidák’s post-test **(A, B)**. Kruskal-Wallis with Dunn´s post-test **(C, D)**. Spearman test **(E)**. *p < 0.05, **p < 0.01, ***p < 0.001, ****p < 0.0001. Color code: previously infected individuals are indicated in red; control TT results are indicated by blue filled circles.

Mean trajectory values of antibody responses and their surrogate neutralizing capacity to SARS-CoV-2 vaccines were assessed in the RTX group (after excluding the two pre-infected patients) and compared with HC and RA group (**Figures 2A, B**). Individual values of all RTX patients (including the two pre-infected patients), RA and HC groups are shown in **Supplementary Figures S1B-D**. All patients showed neutralizing IgG seroconversion 3 weeks after 2^nd^ vaccination (d42). 6 months after 2^nd^ vaccination there was a clear decline of antibody levels and surrogate neutralisation capacity in all cohorts. However, 6 months after 2^nd^ vaccination and d21 after 3^rd^ vaccination, RTX treated patients revealed the lowest surrogate neutralisation capacity compared with HC and RA group (**Figure 2B**). Other than in the RTX group, IgG levels and neutralizing capacity of HC and RA patients on other therapies were significantly boosted upon 3^rd^ vaccination (**Figures 2A, B**). On individual level, 6 months after 2^nd^ vaccination only 3/7 RTX treated patients (including the two previously SARS-CoV-2 infected patients) still revealed neutralizing IgG titres, while two more patients seroconverted upon 3^rd^ vaccination (**Supplementary Figure S2B**). Upon 3^rd^ vaccination we observed significant lower titres while no difference regarding neutralizing capacity among seroconverted RTX patients compared to HC (**Figure 2C**). With regard to steady state control antibodies, anti-TT antibody titers revealed no differences between the groups (**Figure 2D**). In all groups there was a direct correlation between the level of anti-S1 IgG antibodies after 3rd with the one after 2nd vaccination (**Figure 2E**), suggesting that pre-existing memory predicts subsequently humoral response after boost independently upon RTX treatment.

### Diminished circulating total and antigen-specific B cells upon RTX treatment

**Figure 3:**
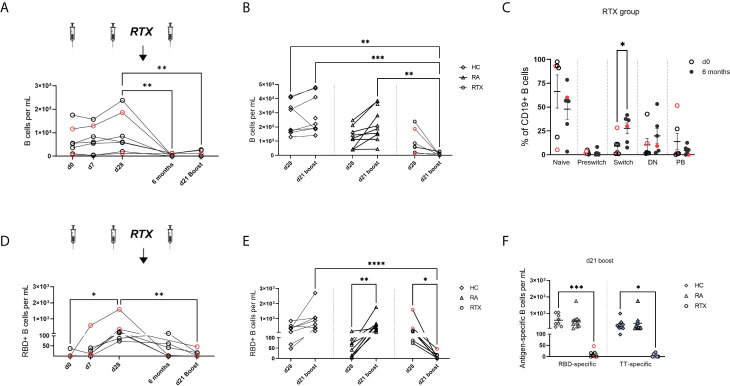
Trajectory of total B cells and antigen-specific B cells upon SARS-CoV-2 vaccination in RTX treated patients. **(A)** Total B cell counts over time in the RTX group (n=7). **(B)** Comparison of total B cell numbers between HC (n=10), RA (n=10) and RTX (n=7) at d28 and d21 boost. **(C)** B cell subset distribution at d0 and 6 months (before 3^rd^ vaccination) in the RTX group (n=7). **(D)** Absolute numbers of RBD+ B cells over time in the RTX group (n=7). **(E)** Absolute numbers of RBD+ cells at d28 compared with d21 boost in HC (n=10), RA (n=10) and RTX (n=7). **(F)** Absolute numbers of RBD+ B cells at d21 boost compared with TT+ B cells in HC, RA and RTX. Friedman test with Dunn´s post-test. **(A, D)**. Kruskal-Wallis with Dunn´s post-test **(B, E, F)**. Mann Whitney test for each subset performed **(C)**. *p < 0.05, **p < 0.01, ***p < 0.001, ****p < 0.0001. Color code: previously infected individuals are indicated in red; control TT results are indicated by blue filled circles. DN, “double negative” CD27+IgD- B cells; PB, plasmablasts.

Total B cell numbers were assessed at different time points in RTX treated patients (**Figure 3A**) and compared with HC and RA after 2^nd^ and 3^rd^ vaccination (**Figure 3B**). As expected, subsequent treatment with rituximab led to a significant decrease of B cell counts compared with the control groups RA and HC (**Figures 3A, B**). Before the 3^rd^ vaccination, naïve B cells had still the highest frequencies of circulating B cells in RTX treated patients. However, we observed an increased frequency of CD27+IgD- switch-memory B cells compared with d0 (**Figure 3C**, gating strategy in **Supplementary Figure S2A**).

Next, we studied SARS-CoV-2 specific B cells over time using flow cytometry to quantify receptor-binding domain (RBD) and as internal control, circulating steady state TT specific B cells (gating strategy in **Supplementary Figure S2A**). In the RTX group the highest RBD+ B cell induction was found at d28 (7 days after 2^nd^ vaccination) compared to lower levels upon RTX treatment and B cell depletion (**Figure 3D**). Other than in RTX group and similar to anti-spike IgG trajectory, RA patients showed a significant enhancement of antigen-specific B cells upon boost vaccination (**Figure 3E**). The number of circulating antigen-specific TT+ B cells was also significantly lower after RTX treatment compared with HC and RA at d21 after boost (**Figure 3F**).

### Induction of IgG+ memory B cells upon 3^rd^ vaccination despite peripheral B cell depletion

**Figure 4:**
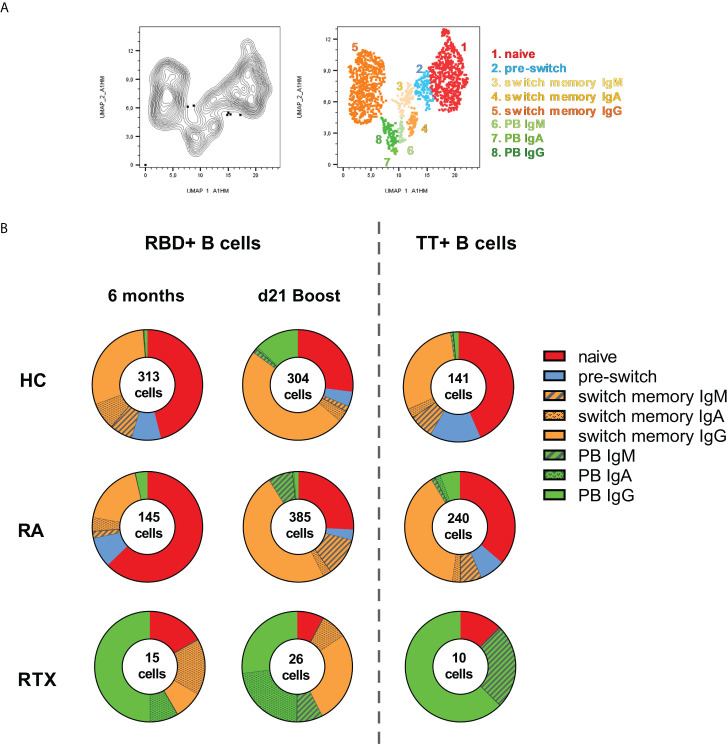
Distinct B cell subsets before and after 3^rd^ SARS-CoV-2 vaccination. UMAP (Uniform manifold approximation and projection for dimension reduction) clustering was performed on a concatenated file of pre-gated antigen specific (RBD+ together with TT+) CD19+ B cells composed of total 1579 events. **(A)** Cluster overlay of B cells of all groups for subset identification. **(B)** Distribution of clusters before and after 3^rd^ vaccination, as well as for TT+ B cells in HD, RA and RTX.

To identify subsets and specific immunoglobulin characteristics of RBD+ B cells, we implemented a high-dimensional flow cytometry analysis of circulating RBD+ B cell subpopulations before and after 3^rd^ vaccination (and as control TT+ B cells at steady state) using Uniform Manifold Approximation and Projection (UMAP). Clusters corresponding to distinct subsets of CD19+ B cells (**Figure 4A**) were defined as: naïve (CD27-IgD+), pre-switch memory (CD27+IgD+), switch memory (CD27+IgD-) and plasmablasts (PB, CD27+CD38+, distribution of key markers shown in **Supplementary Figure 2B**). Switch-memory B cells and PBs clustered according to immunoglobulin specificities IgA, IgM and IgG, respectively. Data of clusters gated in each donor group are shown in **Figure 4B** (individual distribution in **Supplementary Figure S2C**, mean values in **Supplementary Figure S2D**). In HC, RBD+ B cells 6 months after 2^nd^ vaccination and TT+ B cells during steady state showed a similar subset distribution. Pre-switch and switch-memory B cells accounted for more than half of all antigen-specific B cells, followed in frequencies by naïve B cells, while PBs made up less than 2% of circulating antigen-specific B cells. Upon 3^rd^ vaccination there was an enhanced differentiation of RBD+ B cells into IgG+ memory B cells and PBs.

Other than in HC, the majority of RBD+ B cells in RA before 3^rd^ vaccination consisted of naïve cells and RA patients had a higher frequency of circulating TT+ PBs. After 3^rd^ vaccination there was a relevant enhancement of IgG+ switch memory B cells and PBs in RA. However, and distinct to HC, the RA patients showed also a substantial increase in IgM+ PB and switch memory RBD+ B cells, suggesting a delayed immune response in immunosuppressed RA patients (**Figure 4B**).

Relevant differences were found among the RTX patients, although the number of antigen-specific B cells was limited. The predominant subset consisted of plasmablasts: TT+ and RBD+ PBs before vaccination were mostly IgG specific, followed by IgM in TT+ and IgA in RBD+ B cells. Upon 3^rd^ vaccination we observed a relevant increase in IgG+ class-switched memory B cells to the detriment of naïve B cells, but no obvious difference in the magnitude of plasmablasts compared to RBD+ before vaccination and steady state TT+ cells. Of note, only RTX treated patients revealed a high amount of IgA+ memory B cells before 3^rd^ vaccination with induction of IgA+ plasmablasts upon boost, suggesting a persistent but atypical germinal center activity (**Figure 4B**).

### Memory formation and cytokine production of spike-specific T cells is boosted by 3^rd^ vaccination in RTX patients

**Figure 5:**
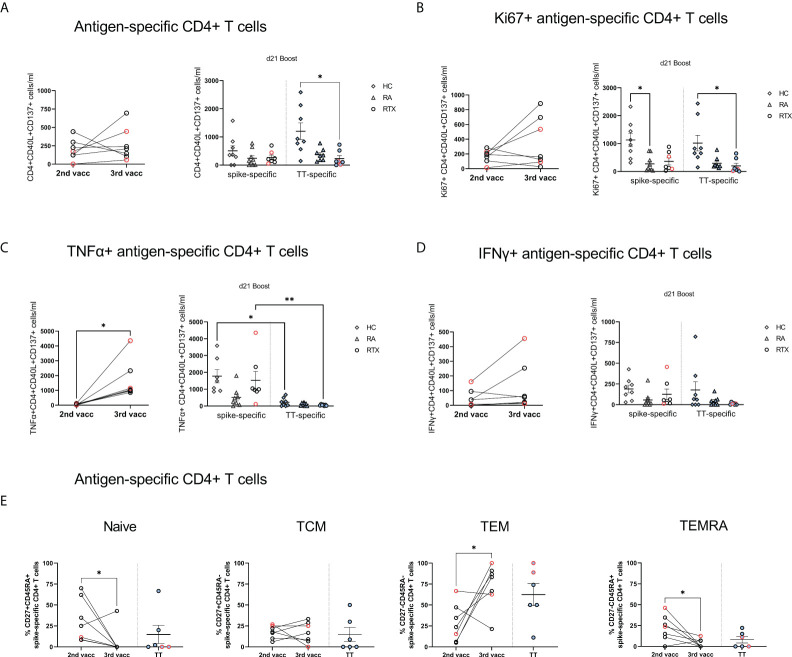
Antigen-specific T cells upon 2^nd^ compared with 3^rd^ SARS-CoV-2 vaccination in RTX treated patients. **(A)** Absolute counts **(A)**, Ki67 expression **(B)**, TNFα expression **(C)**, IFNγ expression **(D)** and subset distribution **(E)** of spike-specific CD4+ T cells after 2^nd^ compared with 3^rd^ vaccination in RTX treated patients (n=7). Comparison of spike-specific and TT-specific responses in HC (n=8), RA (n=8) and RTX (n=7) at d21 boost. Mann Whitney test performed for comparisons between 2^nd^ and 3^rd^ vaccination data **(A-E)**. Kruskal-Wallis with Dunn´s post-test for comparisons between the groups **(A-D)**. *p < 0.05, **p < 0.01. Color code: previously infected individuals are indicated in red; control TT results are indicated by blue filled circles. TCM, central memory T cells; TEM, effector memory T cells; TEMRA, terminally differentiated memory T cells.

Next, we addressed the question if T cell reactivity can be boosted in B cell depleted patients upon 3^rd^ vaccination. Therefore, we assessed induction, memory formation and cytokine production of spike-specific CD4 and CD8 T cells upon 2^nd^ and 3^rd^ vaccination in RTX treated patients. As previously described, antigen-reactive CD4+ T cells were identified based on co-expression of CD154 and CD137, and antigen-reactive CD8+ T cells by co-expression of CD137 and IFNγ ( (15, 19), gating strategy **Supplementary Figures 3A, B**). In a first step, assessment of CD4+, CD8+ and TfH-like (CXCR5+PD1+) total cell numbers did not show any differences between the cohorts (**Supplementary Figure S3C**). While there was also no significant difference regarding total cell counts (**Figure 5A**) and proliferation (**Figure 5B**) of CD4+ antigen-specific T cells between 2^nd^ and 3^rd^ vaccination, we observed a significant increase in TNFα secretion (**Figure 5C**) accompanied by the tendency for higher IFNγ production (**Figure 5D**) of spike-specific CD4 T cells upon boost. Notably, spike-specific effector memory CD4 T cells (TEM, CD27-CD45RA-) were significantly increased upon 3^rd^ vaccination, followed by a concomitant decrease in naïve (CD27+CD45RA+) and terminal differentiated TEMRA (CD27-CD45RA+) CD4 T cell frequencies (**Figure 5E**). When we looked also into the subset distribution upon TNFα and IFNγ secreting spike-specific CD4 T cells, we saw a similar pattern, with highest frequencies upon the TEM subpopulation (**Supplementary Figure S3D**). Analyzing spike-specific CD8+ T cells, we did not observe any difference regarding total numbers, proliferation and subset distribution of antigen-specific CD8+ T cells between 2^nd^ and 3^rd^ vaccination (**Supplementary Figures 3E, F**).

Regarding TT+ T cells, total numbers (**Figure 5A**) and proliferation (**Figure 5B**) of TT+ CD4 cells were significantly lower in RTX patients compared with HC, but similar when compared with RA group, suggesting rather a disease than a therapy specific effect. TNFα-secretion was significantly higher after 3^rd^ SARS-CoV-2 vaccination in HC and RTX groups than upon TT stimulation at steady state (**Figure 5C**), likely related to the more recent vaccine challenge by SARS-CoV-2. Spike-specific and TT-specific CD8+ responses showed similar pattern in all groups.

## Discussion

In the current study we could show that patients re-exposed to RTX after successfully seroconversion upon 2x SARS-CoV-2 vaccination, reveal a persistent but atypical germinal center activity within recall vaccination. Germinal centers are lymphoid structures in which B cells acquire affinity-enhancing somatic hypermutations, differentiating into memory B cells and long-lived bone marrow plasma cells (BMPCs) and providing durable protective immunity upon infection or vaccination (1)). Extrafollicular activation does not seem to play a significant role upon SARS-CoV-2 mRNA vaccination in healthy, but may be part of the dysfunctional immune response upon vaccination in immunocompromised patients (20). With respect to primary 2x anti-SARS-CoV-2 vaccination, we have previously shown that RTX treated patients carrying a minimum of peripheral B cell repopulation, are able to mount antigen-specific MBCs and plasmablasts comparable with HC, suggesting GC formation and possible generation of long-lived MBCs (15). In line with this, recently published data report similar durability of IgG anti-spike antibodies 6 months after primary immunization in seroconverted patients with anti-CD20 treatments compared with healthy volunteers (21). In our RTX cohort we saw persistent antibody titers for TT at steady state and in some patients also for SARS-CoV-2 at 6 months, suggesting the continued presence of long-lived antigen-specific antibody-secreting cells in the bone marrow regardless of peripheral B cell depletion (21–24).

Interestingly, despite lower SARS-CoV-2 antibody level upon 3^rd^ vaccination, neutralization capacity was similar between seroconverted RTX patients and HC. This fine-tuning of high-affinity antibodies appears to be a direct result of somatic hypermutation (25), which seems functional also in RTX treated patients and may be effected either by still available newly recruited peripheral B cells or by preformed MBCs reentering germinal centers. Moreover, we observed a direct correlation between anti-spike SARS-CoV-2 antibody values after 2^nd^ with the ones after 3^rd^ vaccination, suggesting that also upon RTX re-exposure, induction strength of the immune response within recall vaccination, is largely related to pre-existing memory formation. In support of this notion, the highest antibody responses occurred in the two previously infected patients, who may have also mounted stronger germinal centre activation with consecutively long-lived memory.

In line with this, persistent antigen-specific MBCs 6 months after primary immunization could be recorded in the circulation despite RTX treatment, moreover, we observed also an expansion of IgG+ memory B cells upon 3^rd^ vaccination. However, the majority of RBD+ and TT+ B cells in RTX treated patients consisted of IgG+ plasmablasts, which may have escaped depletion due to low CD20 expression or have been recently induced in lymphoid tissues without detectable precursors. Notably, only RTX treated patients revealed a high amount of IgA+ memory B cells before 3^rd^ vaccination with induction of IgA+ plasmablasts upon boost. A subset of circulating IgA+ plasmablasts and plasma cells with mucosal characteristics has been previously described to be resistant to B cell depletion upon RTX treatment (22). Moreover, in healthy volunteers, transient IgA-dominant plasmablast response to the BNT162b2 mRNA vaccine is recently reported predominantly after the first vaccination dose and is consistent with a cross-reactive recall response of mucosal MBCs (26). The induction of IgA+ plasmablasts upon boost observed only in the context of limited peripheral B cell availability suggests atypical B cell mobilization from either tissue resident cross-reactive MBCs and/or within naïve mucosal lymphoid follicles/extrafollicular resident B cells. This disturbed GC reaction seems independent upon T cell functions, since in RTX patients we observed significant boosted cytokine secretion and differentiation to effector memory spike-specific CD4 T cells as well as TfH cell counts comparable with HC within 3^rd^ vaccination.

Our data provide also interesting insights about the delayed GC immune response in RA patients on therapies other than RTX. 6 months after the second vaccination the majority of RBD+ B cells in RA consisted of naïve cells, suggesting an insufficient GC activation after the first two injections lacking long-term memory. Upon 3^rd^ vaccination, RA showed a significant boost of antibody levels and circulating RBD+ B cells in parallel with induction of IgG+ RBD+ memory B cells and plasmablast differentiation comparable with the HC group. Nevertheless, other than in HC, RBD+ memory B cells and plasmablasts of RA patients showed also a substantial increase in IgM+ expression upon boost, reflecting a delayed isotype switching in this immunosuppressed population.

The main limitation of the study is the low patient number and limited B cell counts in the RTX cohort included into the analysis. Two of the RTX patients got previously infected and may have mounted stronger long-lived immune responses, which may be a confounder in the analysis. However, we provide a comprehensive data set regarding trajectories of humoral and cellular B and T cell responses over time including steady state findings of TT. Noteworthy, 5/7 of the RTX treated patients did not have other additional immunosuppressive drugs, which excludes co-medication as confounding factor.

Herein, we describe for the first time humoral and cellular responses upon 3^rd^ SARS-CoV-2 injection in patients re-exposed to RTX after initially seroconversion upon primary vaccination. While functional aspects of spike-specific CD4 T cells are boosted upon 3^rd^ vaccination, we report a persistent but atypical germinal center activity, possibly supported by (semi-primary and/or) additional extrafollicular responses in patients re-exposed to RTX as potential compensatory mechanisms employed in such medically induced B cell impairment.

## Data availability statement

The raw data supporting the conclusions of this article will be made available by the authors, without undue reservation.

## Ethics statement

The studies involving human participants were reviewed and approved by the ethics committee at the Charité University Hospital Berlin (EA2/010/21, EA4/188/20). The patients/participants provided their written informed consent to participate in this study.

## Author contributions

The concept of the study was developed by A-LS, ACL, and TD. Patient’s samples were collected by KK, A-LS and JR. Data were obtained by A-LS, HR-A, FS, JR, YC, BJ, HS and CL. Data were analyzed by A-LS, HR-A and ACL. The theoretical framework was developed by TD, ES, A-LS and ACL. The work was supervised by ACL and TD. All authors developed, read, and approved the current manuscript.

## Funding

A-LS is funded by a grant from the German Society of Rheumatology. HR-A holds a scholarship of the COLCIENCIAS scholarship No. 727, 2015. ES received a grant from the Federal Ministry of Education and Research (BMBF) (BCOVIT, 01KI20161). ES is participant in the BIH-Charité Clinician Scientist Program funded by the Charité Universitätsmedizin Berlin and the Berlin Institute of Health. JR is supported by a MD scholarship from the Berlin Institute of Health (BIH). HS received funding from the Ministry for Science, Research and Arts of Baden-Württemberg, Germany (CORE-Project) and the European Commission (HORIZON2020 Project SUPPORT-E, no. 101015756). YC is supported by a state scholarship fund organized by China Scholarship Council. TD received funding by the German Research Foundation (DFG) by projects TRR 130/project 24, Do491/7-5, Do 491/10-1.

## Conflict of interest

The authors declare that the research was conducted in the absence of any commercial or financial relationships that could be construed as a potential conflict of interest.

## Publisher’s note

All claims expressed in this article are solely those of the authors and do not necessarily represent those of their affiliated organizations, or those of the publisher, the editors and the reviewers. Any product that may be evaluated in this article, or claim that may be made by its manufacturer, is not guaranteed or endorsed by the publisher.
