# Supplementary material for: Persistent but atypical germinal center reaction among 3rd SARS-CoV-2 vaccination after rituximab exposure
Source: Front Immunol. 2022 Aug 10;13:943476. doi: 10.3389/fimmu.2022.943476 (PMC9399943; doi:10.3389/fimmu.2022.943476)
Supplement: Supplementary file 1 [file DataSheet_1.docx]

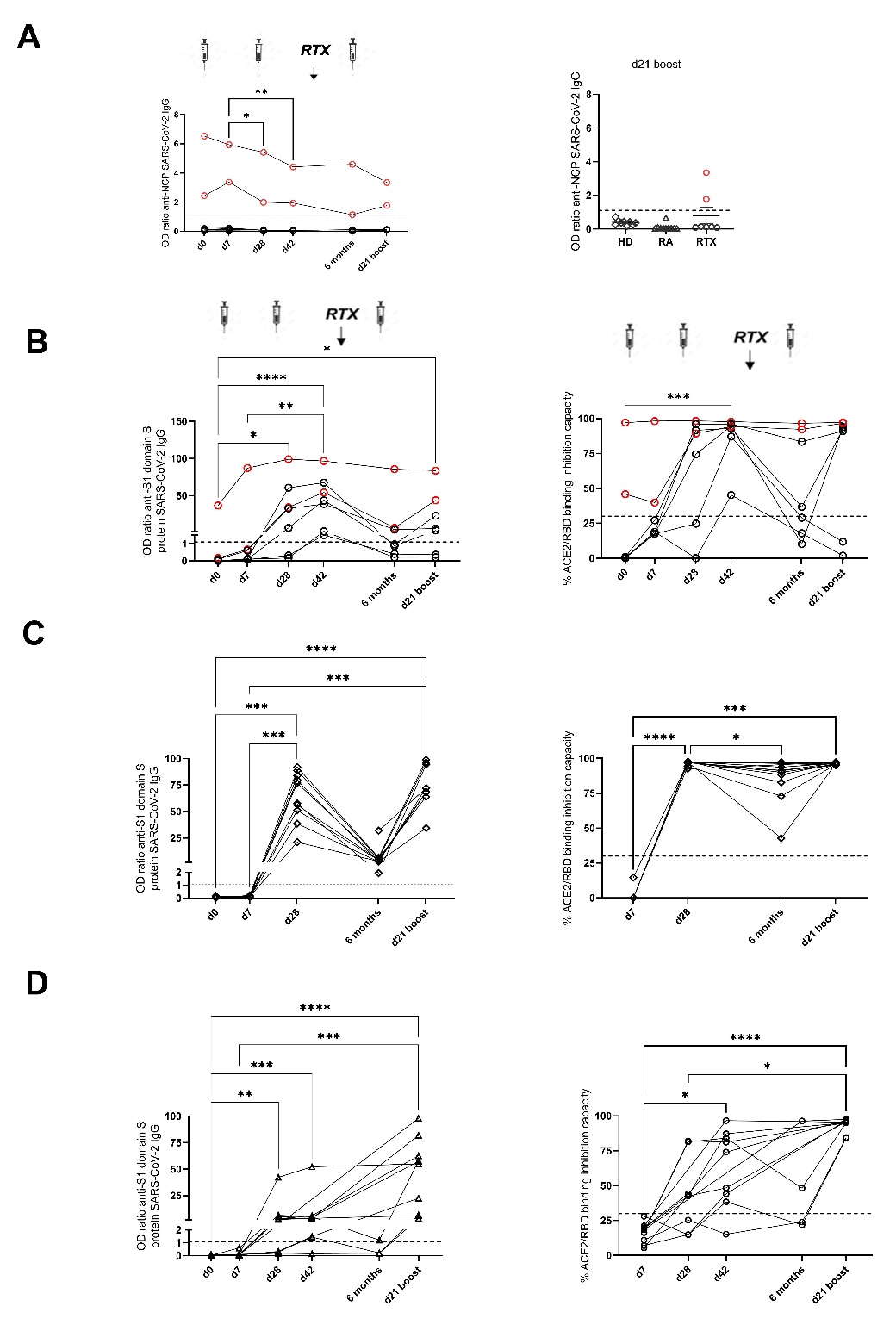


Suppl. Fig. 1. Trajectory of anti-NCP IgG in RTX patients (n=7) at different time points and comparison with HC (n=10) and RA (n=10) at d21 boost (A). Anti-S1 SARS-CoV-2 IgG titers and neutralization capacity in RTX (B), HC (C) and RA group (D). Friedman test with Dunn´s post-test (A-D). Kruskal-Wallis with Dunn´s post-test (A, d21 boost comparison). *p<0.05, **p<0.01, ***p<0.001, ****p<0.0001.


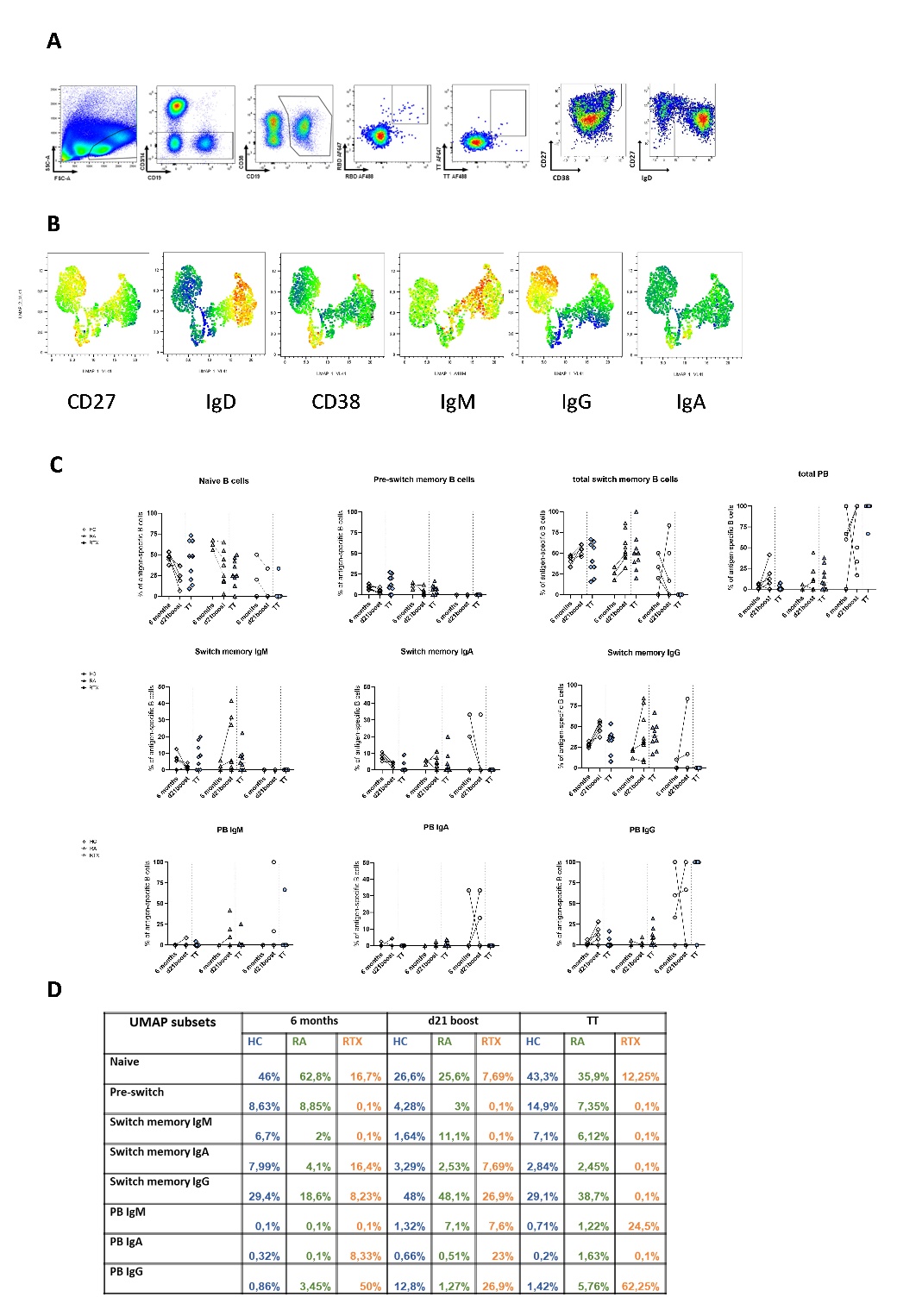


Suppl. Fig. 2. (A) Representative plots of RBD+/TT+ B cells, plasmablasts, and non-plasmablast B cell subsets based on IgD/CD27 classification. (B) Distribution of key markers used for defining UMAP cluster (CD27, IgD, CD38, IgM, IgG, IgA). (C) Individual distribution of B lineage cell clusters according UMAP in HD, RA and RTX. TT+ B cells are indicated by blue filled circles. (D) Mean values of UMAP cluster distribution at 6 months and d21 boost in HC, RA and RTX (as illustrated in Fig. 5B).


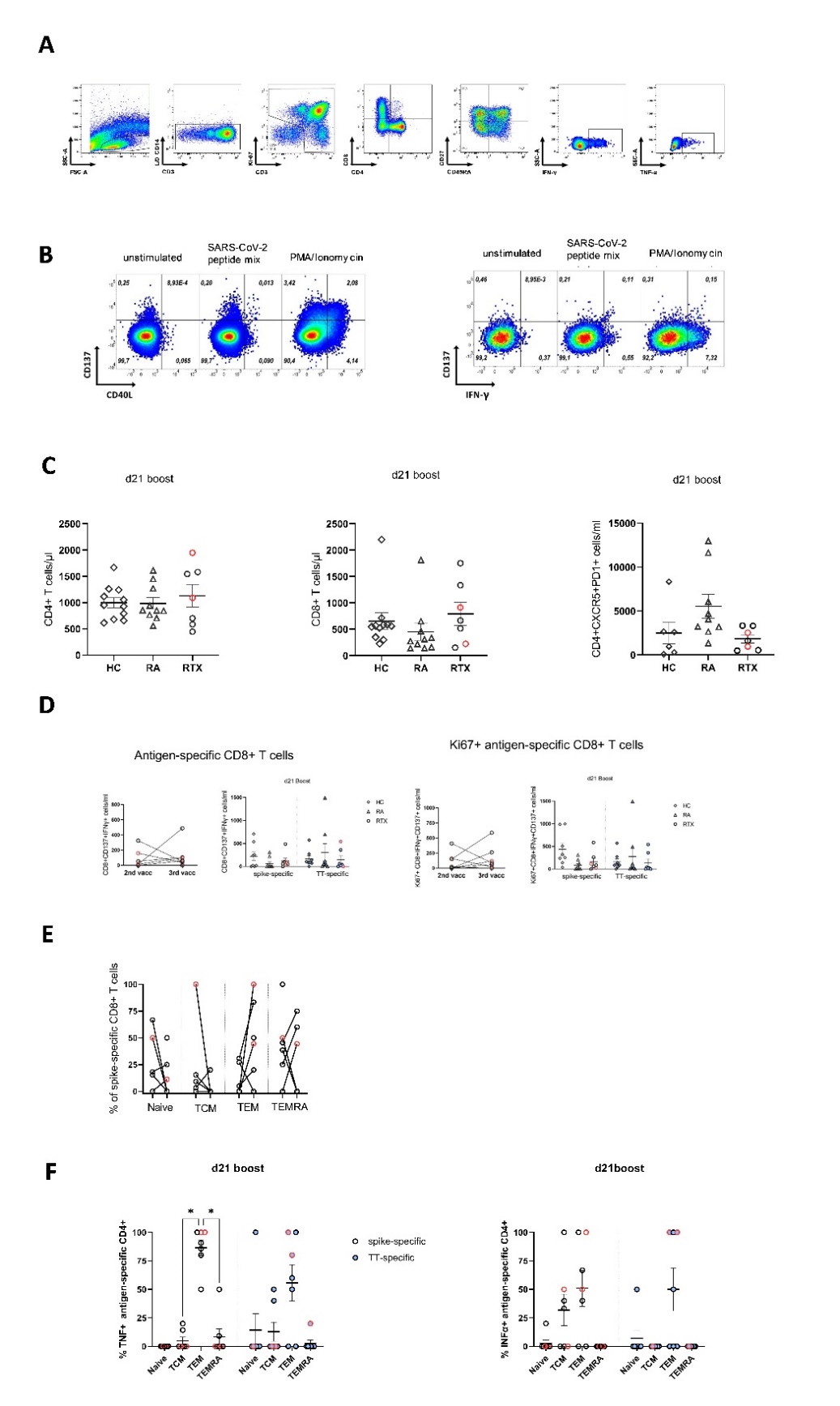


Suppl. Fig. 3. (A) Representative plots of CD4+ and CD8+ T cells and cell subsets based on CD27/CD45RA classification. (B) Representative plots for antigen-specific CD4 T cells (CD137+CD40L+) and CD8 T cells (CD137+IFNγ+) after peptide stimulation compared with no stimulation and stimulation with PMA/Ionomycin. (C) Absolute counts of CD4+, CD8+ and TfH-like (CD4+CXCR5+PD1+) T cells in HC, RA and RTX groups. (D) Absolute counts and Ki67 expression of spike-specific CD8+ T cells after 2nd compared with 3rd vaccination in RTX treated patients (n=7). Comparison of spike-specific and TT-specific responses in HC (n=8), RA (n=8) and RTX (n=7) at d21 boost. (E) Subset distribution among spike-specific CD8+ T cells in RTX treated patients. (F) Subset distribution of TNFα+ and IFNγ+ expressing spike-specific CD4+Tcells in RTX at d21boost. Mann Whitney test performed for comparisons between 2nd and 3rd vaccination data (D). Kruskal-Wallis with Dunn´s post-test for comparisons between the groups (C, D, F). *p<0.05.
